# Effect of organic photovoltaic and red-foil transmittance on yield, growth and photosynthesis of two spinach genotypes under field and greenhouse conditions

**DOI:** 10.1007/s11120-023-01028-8

**Published:** 2023-06-14

**Authors:** Uchenna Noble Ukwu, Joy Udoka Agbo, Onno Muller, Silvia Schrey, Ladislav Nedbal, Yuxi Niu, Matthias Meier-Grüll, Michael Uguru

**Affiliations:** 1 Institute of Bio-and Geosciences, Plant Sciences, Forschungszentrum Julich GmbH, IBG-2, 52428 Julich, Germany; 2 Department of Crop Science, Faculty of Agriculture, University of Nigeria, Nsukka, Nigeria

**Keywords:** Agrophotovoltaics, Photovoltaic transmittance, Photochemical yield, Non-photochemical energy losses, Red and blue light, Shading effect

## Abstract

The galloping rise in global population in recent years and the accompanying increase in food and energy demands has created land use crisis between food and energy production, and eventual loss of agricultural lands to the more lucrative photovoltaics (PV) energy production. This experiment was carried out to investigate the effect of organic photovoltaics (OPV) and red-foil (RF) transmittance on growth, yield, photosynthesis and SPAD value of spinach under greenhouse and field conditions. Three OPV levels (P0: control; P1: transmittance peak of 0.11 in blue light (BL) and 0.64 in red light (RL); P2: transmittance peak of 0.09 in BL and 0.11 in RL) and two spinach genotypes (bufflehead, eland) were combined in a 3 × 2 factorial arrangement in a completely randomized design with 4 replications in the greenhouse, while two RF levels (RF0: control; RF1: transmittance peak of 0.01 in BL and 0.89 in RL) and two spinach genotypes were combined in a 2 × 2 factorial in randomized complete block design with four replications in the field. Data were collected on growth, yield, photosynthesis and chlorophyll content. Analysis of variance (ANOVA) showed significant reduction in shoot weight and total biomass of spinach grown under very low light intensities as a function of the transmittance properties of the OPV cell used (P2). P1 competed comparably (*p* > 0.05) with control in most growth and yield traits measured. In addition, shoot to root distribution was higher in P1 than control. RF reduced shoot and total biomass production of spinach in the field due to its inability to transmit other spectra of light. OPV-RF transmittance did not affect plant height (PH), leaf number (LN), and SPAD value but leaf area (LA) was highest in P2. Photochemical energy conversion was higher in P1, P2 and RF1 in contrast to control due to lower levels of non-photochemical energy losses through the Y(NO) and Y(NPQ) pathways. Photo-irradiance curves showed that plants grown under reduced light (P2) did not efficiently manage excess light when exposed to high light intensities. Bufflehead genotype showed superior growth and yield traits than eland across OPV and RF levels. It is therefore recommended that OPV cells with transmittance properties greater than or equal to 11% in BL and 64% in RL be used in APV systems for improved photochemical and land use efficiency.

**Supplementary Information:**

The online version contains supplementary material available at 10.1007/s11120-023-01028-8.

## Introduction

The push for clean, sustainable and environmental friendly sources of renewable energy as a consequence of climate change, and the bid to meet the global energy demand have led to increased use of solar energy or PV panels in homes and offices. This trend has led to land use crisis between food production and energy (Weselek et al. 2019) as PV installations on open areas remains the most cost effective option (Fraunhofer ISE 2015). There is a growing concern about the loss of agricultural land to the more lucrative PV energy production. Unfortunately, with the global population projected to rise from its current 7.7 billion to 8.5 billion in 2030 (UN Report 2020), the competition for limited agricultural land is expected to rise with greater impacts in very densely populated regions with small land area.

Agrophotovoltaics (APV) is an emerging technology that integrates food production under PV panels (Trommsdorff et al. 2021). Organic photovoltaic (OPV) cells are small, flexible and semi-transparent type of PVs that uses organic semi-conductors to produce electricity from sunlight (Cheng et al. 2018; Hou et al. 2018). It has absorption properties that can be modified to complement the light spectral range (400–700 nm) of photosynthesizing plants (Ravishankar et al. 2021) while selectively using irradiance outside the plant light range for electricity production. Due to its small sizes, it can be installed over plants in limited space. APV has the potential to provide the right balance between food and energy needs of the future populace (Ravishankar et al. 2021), increase land use efficiency by up to 80% (Touil et al. 2021), reduce photodamage by cutting the proportion of irradiance reaching the plants by up to 40% (Allardyce et al. 2017; Dijk et al. 2021), and increase efficiency of photosystem II (PSII) photochemistry (Ravishankar et al. 2021) which is directly correlated with yield (Xu et al. 2020). In addition, electricity produced from the PV cells can be used to power farm appliances, and the shading effect can help conserve water during dry spells which are invaluable cost saving benefits to a farmer.

Spinach (*Spinacia oleracea*) is a very nutritious leafy vegetable of the amaranth family with huge potentials to thrive in shade and extreme cold conditions in addition to its short life cycle. The objectives of this study were to determine the effects of integrating spinach under OPV cells (in greenhouse) or simulating red foils (RF) (in field) on growth and yield performance, photosynthesis and chlorophyll content of two spinach genotypes in greenhouse and field conditions during the winter season.

Recent studies involving APV have been done using opaque panels and largely on PV cover ratios. For instance, Ezzaeri et al. (2018) observed similar growth and yield patterns in shaded and control treatments when tomato was grown under 10% PV cover ratio; Liu et al. (2019) reported comparable stem length in mungbean grown under PV shading; Zisis et al. (2019) reported 21.8% taller plants in pepper grown under PV with 22% cover ratio; Tang et al. (2020) observed 30% and 20% increase in chlorophyll content and fruit weight, respectively for PV shaded strawberry at 25.9% cover ratio relative to control; Thompson et al. (2020) reported 14 and 53% increase in protein content of basil and spinach, respectively for PV shaded treatment compared to control, and up to 68% in pepper in addition to improved photosynthetic efficiency (Ravishankar et al. 2021); Hassanien et al. (2022) reported taller plants with higher fruit yields (*p* > 0.05) in PV shaded (13–26% cover ratio) chili pepper compared to control.

This study provides the first report on APV using OPV cells and RF in literature. We hypothesized that growing spinach under OPV cells or RF could produce comparable yield under strong light intensities to growing them in open field. We investigated the response of two spinach varieties to different light transmittance provided by two OPV cells and an RF. We constructed photo-irradiance curves over a wide internal PPFD range to ascertain how increasing light intensities and quality of light transmitted interact. We quantified photosynthesis, SPAD value, and growth indices over a less variable sun-induced PPFD range to understand how these variables were affected by transmittance quality and genotype.

## Materials and methods

### Experimental site

Two experiments were carried out at the Institute of Bio-and Geosciences (IBG-2), Plant Sciences, Forschungszentrum Julich GmbH, 50.9096° N, 6.4130° E, Germany. The field experiment was carried out at the research garden of IBG-2 while the greenhouse experiment was carried out at the greenhouse of IBG-2 between October 2021 and February 2022.

### Materials

**Fig. 1:**
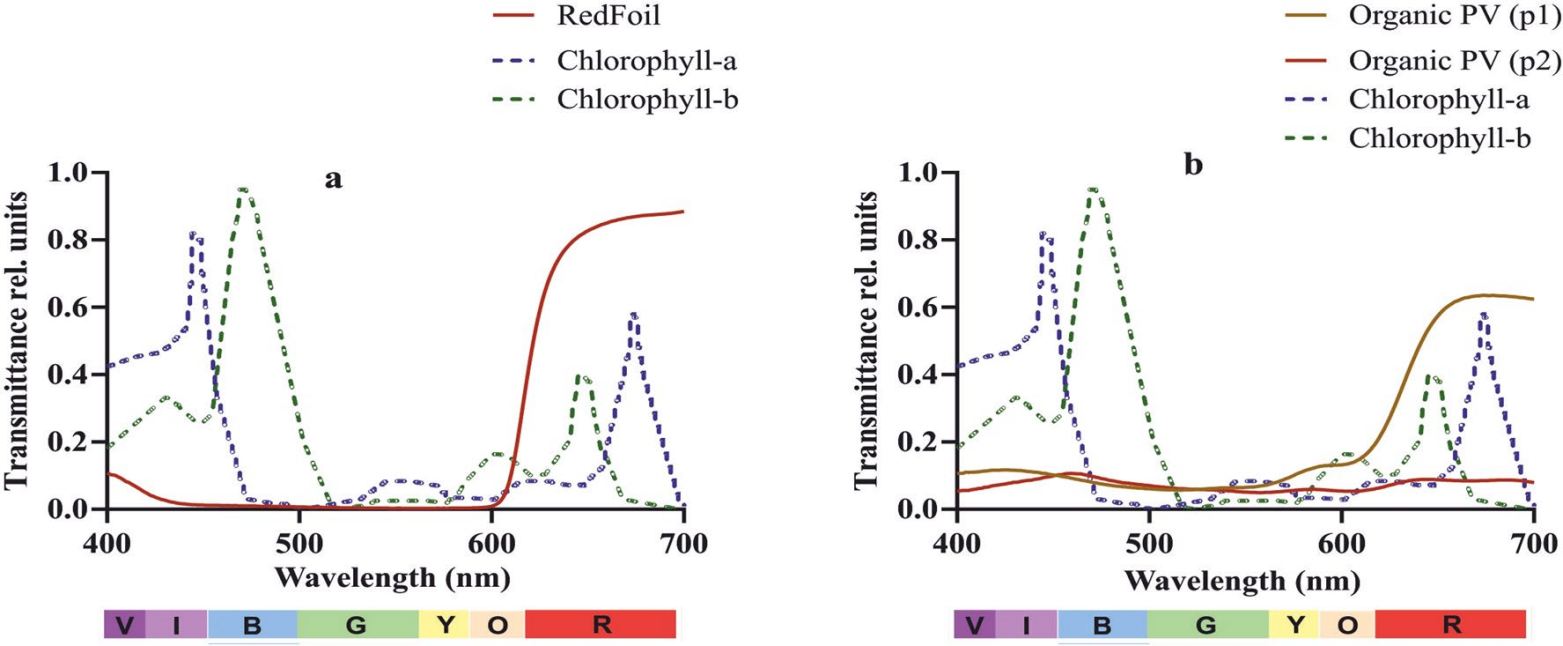
Transmittance Properties of Red-Foil (**a**) and Two Organic PVs (**b**) with Absorption Spectra of Chlorophyll *a* and *b* in Dotted Lines

Two spinach (*Spinacia oleracea*) genotypes, Bufflehead (shade-loving) and Eland (shade-shy) were selected for the experiment due to their distinct behavior when grown under shaded environment. Two OPV cells, P1 with transmittance peaks of 0.11 and 0.64, and P2 with transmittance peaks of 0.09 and 0.11, in blue and red lights, respectively (Fig. 1a), were used for the greenhouse experiment while a simulating RF with transmittance peaks of 0.01 and 0.89 in blue and red lights (Fig. 1b) were used at the field for comparison as the OPV cells were too small to be installed over plants on the field. Einheitserde Classic Pikier substrate, Metrob, Slovenia (a commercial product) was used as the growth medium and was filled in 1.5-L pots in the greenhouse. Mini-PAM-II-device (Heinz Walz, Germany) was used for photosynthetic yield analysis and SPAD (Konica Minolta, GmbH, Germany) device was used for determination of relative chlorophyll value.

### Experimental design, plant establishment and OPV-RF installations

The experimental design for the greenhouse was a 3 × 2 factorial in CRD replicated four times while the design for the field experiment was a 2 × 2 factorial in RCBD replicated four times.

In the greenhouse, seeds were sown in germination trays prefilled with pikier substrate, and seedlings with uniform emergence after 7 days were transplanted into the pots. 100 ml of water was applied once in 2 days with greenhouse temperature fixed at 15 °C. At the field, seeds were sown directly on flat at a spacing of 40 × 30 cm with a gap of 50 cm between plots. No fertilizer was applied. Each plot size measured 0.48 m^2^. The field was irrigated every two days by the aid of a watering-can on days it did not rain. The field was kept weed free manually by use of hoe. Both greenhouse and field plants were closely monitored.

**Plate 1:**
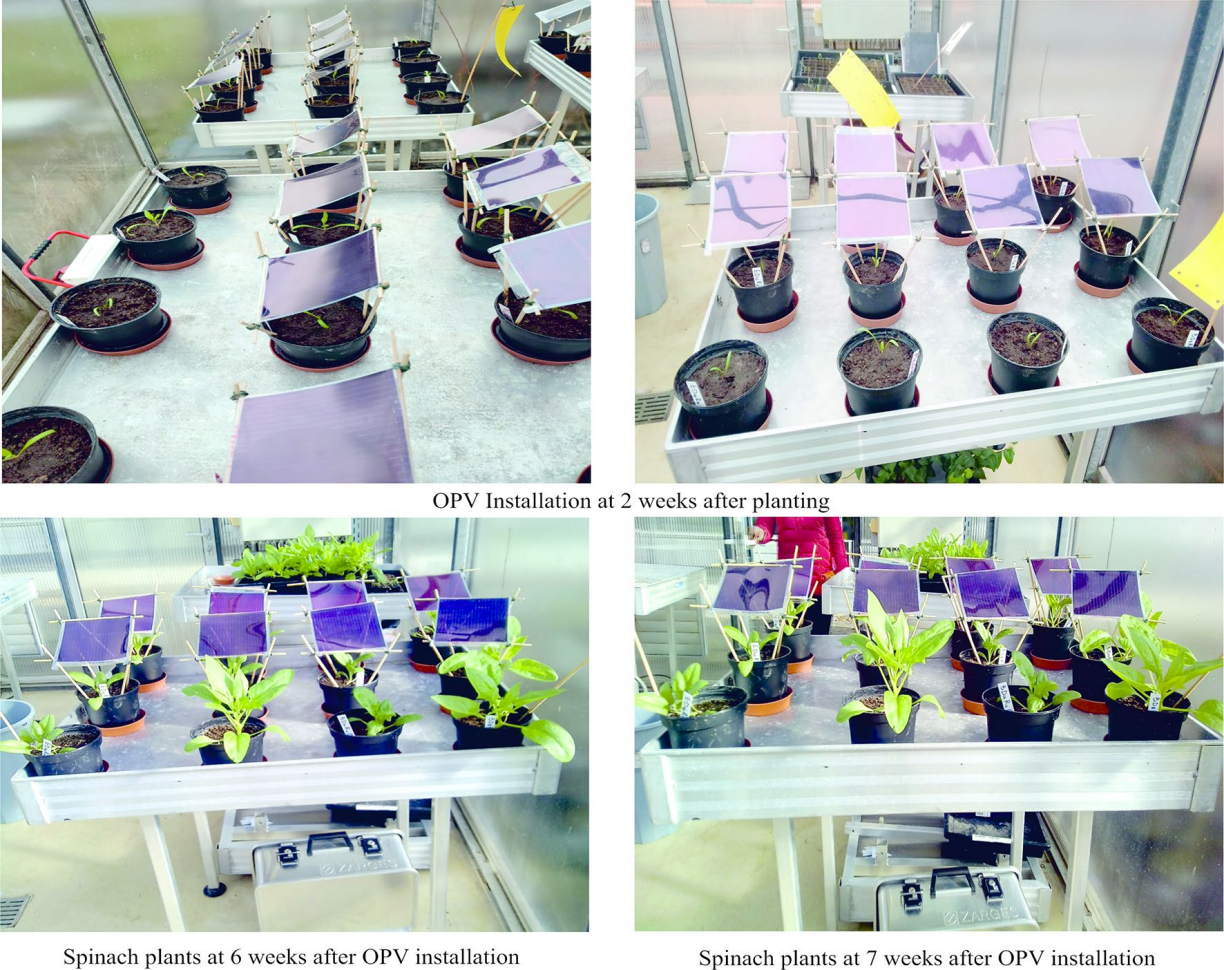
Two spinach genotypes growing under two OPV cells (P1, P2) and no OPV (P0) in a 3 × 2 factorial combination. Each section of the plate show pictures of two replicates

**Plate 2:**
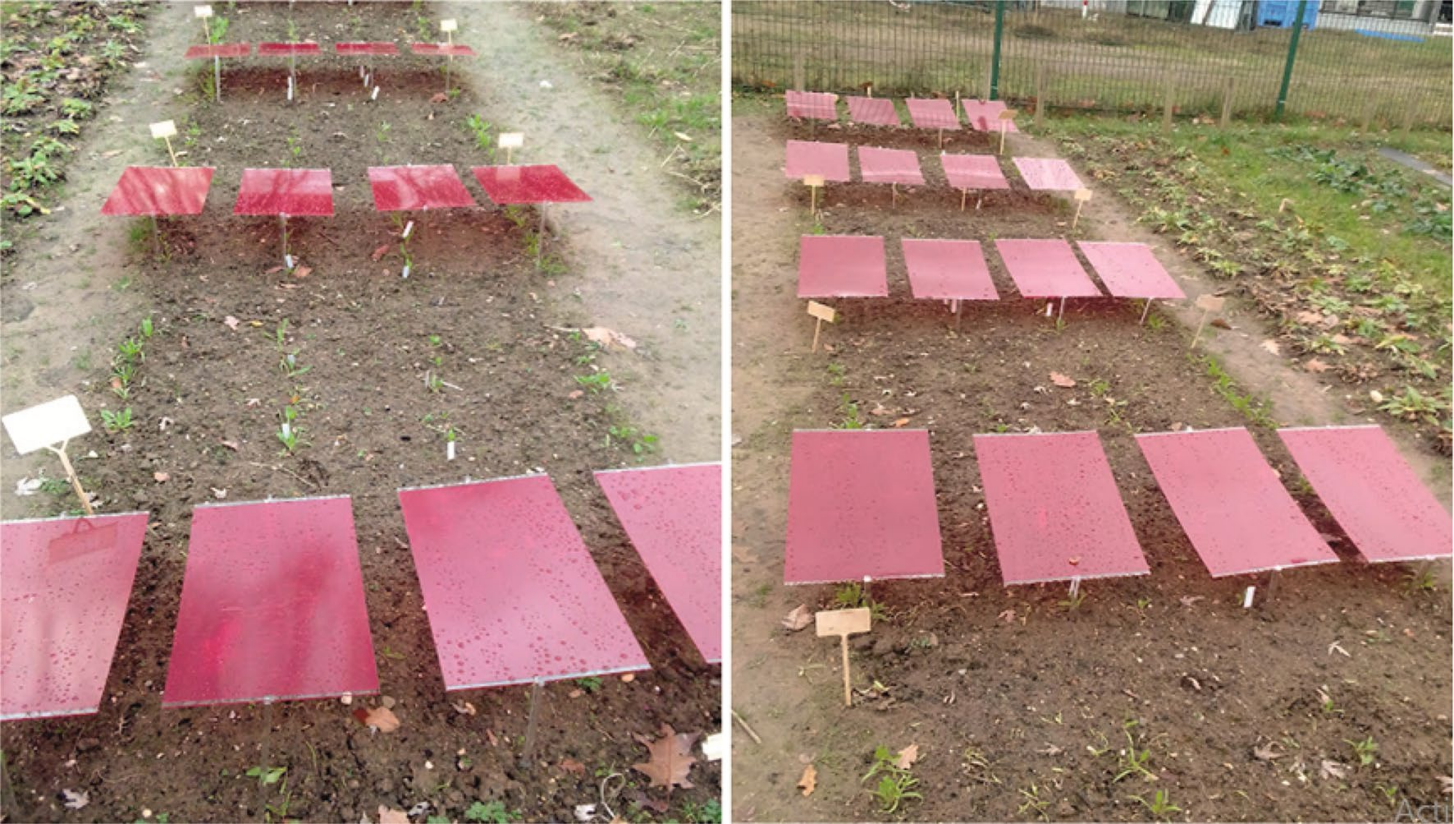
Two spinach genotypes growing under red foil sheet (RF1) and no red foil (RF0) in a 2 × 2 factorial combination in the field

Two OPV cells (Greenhouse-sheets) of dimension 30 × 30 cm were installed over the plants at 45° to the sun and at a height of 45 cm from the base of the medium two weeks after transplanting in the greenhouse (Plate 1). RFs were installed over the plants using the same dimension as in the greenhouse six weeks after planting following very slow growth rate occasioned by extreme cold during winter (Plate 2).

### Data collection

Data were collected on absolute fluorescence traits such as minimum and maximum fluorescence of dark acclimated leaves (F_0_, Fm), minimum and maximum fluorescence of illuminated leaves (F_0_', Fm'), and transient fluorescence level (F') which were used to compute photosynthetic parameters including maximum photochemical yield (Fv/Fm), effective photochemical yield of photosystem II (PSII) Y(II), quantum yield of non-photochemical energy losses via heat dissipation at the antenna Y(NPQ), quantum yield of non-photochemical energy losses via heat dissipation and fluorescence at the reaction centers, and electron transport rate (ETR).

Data were also collected on SPAD chlorophyll value, growth and yield traits such as plant height (PH), leaf number (LN), leaf area (LA), fresh shoot weight (FSW), dry shoot weight (DSW), dry root weight (DRW), Total biomass weight (TBW), leaf mass per area (LMA) and total energy distribution (TED).

### Measurement of growth and yield traits

PH was determined using a meter rule; LN was counted as the number of fully expanded leaves per plant; LA was determined as the average of three to five leaves per plant using the L1-3100 Area Meter; FSW was determined as the fresh weight of shoot per plant after separation of root; DSW was determined as the weight of shoot per plant after oven-drying to a constant weight; DRW was determined as the weight of root per plant after oven-drying to a constant weight; TBW was determined as the sum of shoot and root weight per plant; TED was determined as the ratio of shoot to root weight per plant while LMA was determined as the leaf area per gram of plant.

### Measurement of photosynthesis parameters

To evaluate the photosynthetic efficiency of light accounted for by RF and two OPV cells, spinach plants were exposed to 12 photosynthetic photon flux density (PPFD) levels ranging from 25, 45, 65, 90, 125, 190, 285, 420, 625, 820, 1150 and 1500 µmol m^−2^ s^−1^ for photo-irradiance response curves. Greenhouse plants were moved to a dark room where they were dark adapted for 30 min to measure Fv/Fm. Dark leaf clips were used to achieve the same objective for plants growing in the field. Weekly measurements were made on third and fourth leaves from the top per plant under natural light conditions between 10.00 and 11.30 am and intensity of 120–200 µmol m^−2^ s^−1^ using a portable pulse amplitude-modulated chlorophyll fluorometer (Mini-PAM II) at a measuring light intensity of about 0.04 µmol m^−2^ s^−1^ and a saturating light pulse of about 5000 µmol m^−2^ s^−1^. Y(II), Y(NPQ), Y(NO) and ETR were computed following the procedures of Genty et al. (1989, 1996) as follows:

Y(II) = (Fm' – F')/Fm', Y(NO) = F'/Fm, Y(NPQ) = (F'/Fm') – (F'/Fm), ETR = PAR × ETR factor × (P_PS2_/P_PS1+2_) × Y(II). Where PAR is photosynthetic active radiation, ETR factor and (P_PS2_/P_PS1+2_) are constants with values of 0.84 and 0.5, respectively.

Y(II), Y(NPQ) and Y(NO) are three complementary pathways through which absorbed excitation energy are relaxed in PSII, and the three sum up to one (Genty et al. 1996). These traits are fundamental in partitioning the proportion of absorbed energy that is actually used for PSII photochemistry, the proportion of excess excitation energy that is harmlessly dissipated at the antenna, and the proportion of excess excitation energy that reaches reaction centers (RC) which could potentially cause photodamage under very high intensity. For instance, at very high irradiance, higher Y(NPQ) values relative to Y(NO) implies that excess light were successfully dissipated at the antenna before reaching the RC and that photosynthetic fluxes are well regulated. In contrast, higher Y(NO) over Y(NPQ) is an indication that excess light is reaching the RC causing strong reduction in PSII acceptors and photodamage (Genty et al. 1996). It could also suggest that both photochemical energy conversion and protective regulatory mechanisms are inefficient, thus indicative that the plant had difficulty coping with the incident radiation (Huang et al. 2011).

### Measurement of RF and OPV transmittance

To establish the spectra range of light transmitted by the RF and OPV materials, the materials were passed through a high resolution ASD FieldSpec 4 (Malvern Panalytical) following the procedure: (i) optimize integration time; (ii) collect white reference (WR) with fiber facing sphere opening and lamp on; (iii) hold front of sample in front of sphere opening and ensure no light is escaping to the sides, take measurement; (iv) hold back of sample in front of sphere opening and ensure no light is escaping to the sides, take measurement; (v) repeat iii and iv for all samples.

At the end of the measurement, RF had the least transmittance (0.01) of BL while P1 and P2 had relatively higher transmittance (0.11 and 0.09, respectively). For RL transmittance however, RF was highest (0.89) followed by P1 (0.64) and P2 (0.09) (Fig. 1).

### Statistical analysis

To test for significance of treatment means, analysis of variance (ANOVA) was carried out for all data using GenStat 10.3 discovery edition, and post-ANOVA mean separation was achieved using least significant difference (LSD) at *p* < 0.05. GraphPad Prism 9 was used to produce the graphs.

## Results

### Effect of OPV-RF transmittance, genotype and interaction on yield and yield attributes of spinach in greenhouse and field conditions

**Fig. 2:**
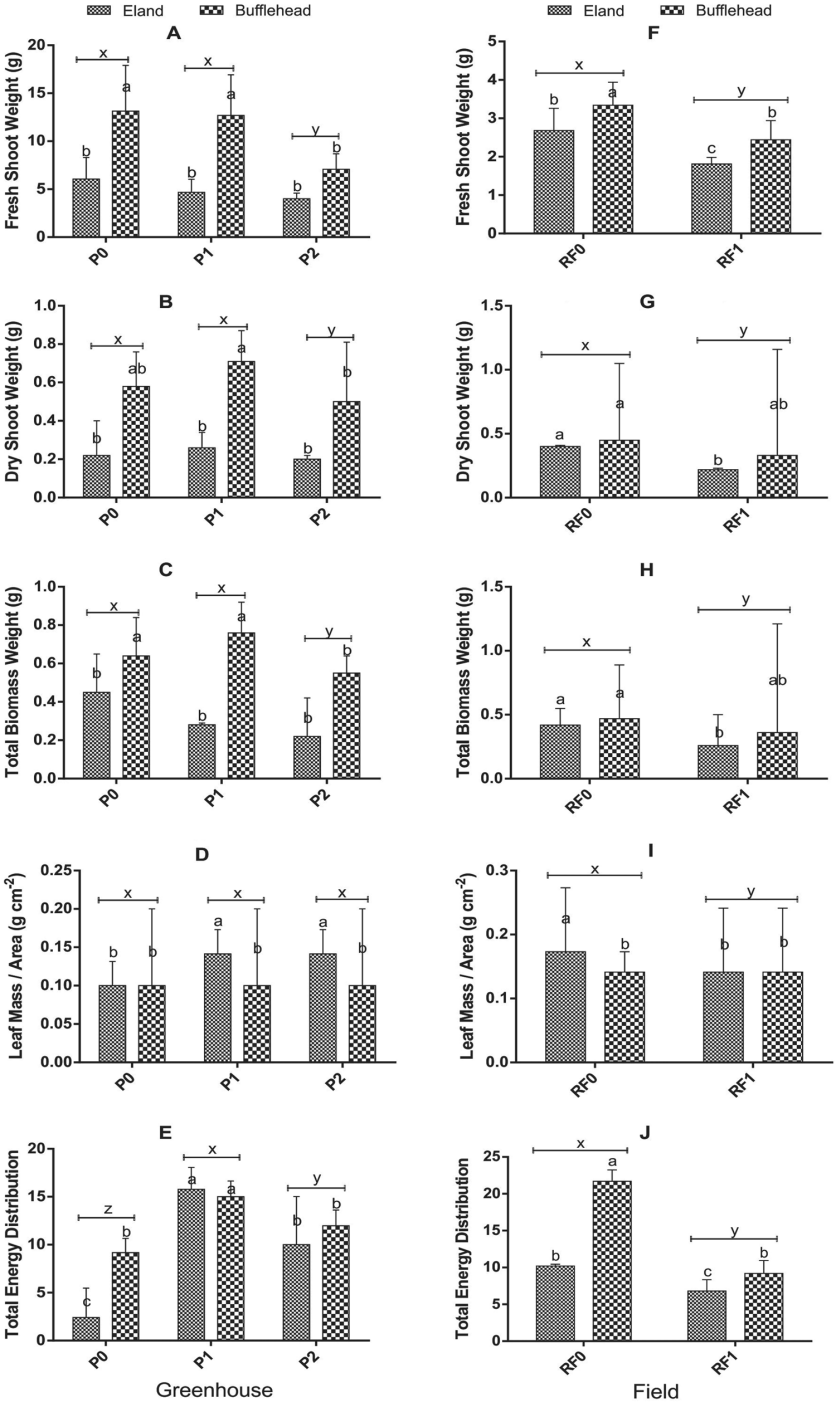
Effect of OPV-RF Transmittance, Genotype and Interaction on Leaf Yield and Yield Traits of Spinach in Greenhouse and Field Conditions. Bars show mean and standard deviations. Bars with different letters are significantly different at *p* < 0.05. P0: Plants grown under no OPV in greenhouse; P1: plants grown under OPV with transmittance peak of 0.64; P2: plants grown under OPV with transmittance peak of 0.11; RF0: plants grown under no red-foil in the field; RF1: plants grown under red-foil with transmittance peak of 0.89

The effect of OPV-transmittance on leaf yield and yield attributes of spinach in greenhouse and field conditions are shown in Fig. 2. OPV-transmittance significantly influenced FSW, DSW and TED (*p* ≤ 0.05) but did not affect LMA in the greenhouse (Fig. 2A–D). P0 and P1 were similar in FSW and DSW and were higher than P2. TED was higher in P1 (15.27 ± 2.29) and P2 (10.99 ± 3.62) than P0 (5.79 ± 4.25) (Fig. 2E). The effect of RF on yield and yield attributes of spinach was significant (*p* < 0.05) for FSW, DSW, TBW, LMA and TED in the field (Fig. 2F–J). RF0 was significantly higher than RF1 in all five yield traits.

There was a clear case of genotypic effect in almost all the yield traits measured. With the exception of LMA (Fig. 2D and I) where eland was superior to bufflehead, bufflehead genotype was consistently higher in FSW, DSW, TBW and TED than eland in greenhouse and field (Fig. 2).

Significant OPV-genotype interaction was recorded in FSW, DSW and TBW in the greenhouse. Bufflehead grown in open (BP0) and bufflehead grown under P1 (BP1) had superior FSW, DSW and TBW than the others. Interaction effect on LMA and TED were not significant (Fig. 2A–E). Likewise, the effect of RF-genotype interaction did not significantly affect yield attributes of spinach in the field (Fig. 2F–J).

### Effect of OPV-RF transmittance, genotype and interaction on growth and SPAD value of spinach in greenhouse and field conditions

**Fig. 3:**
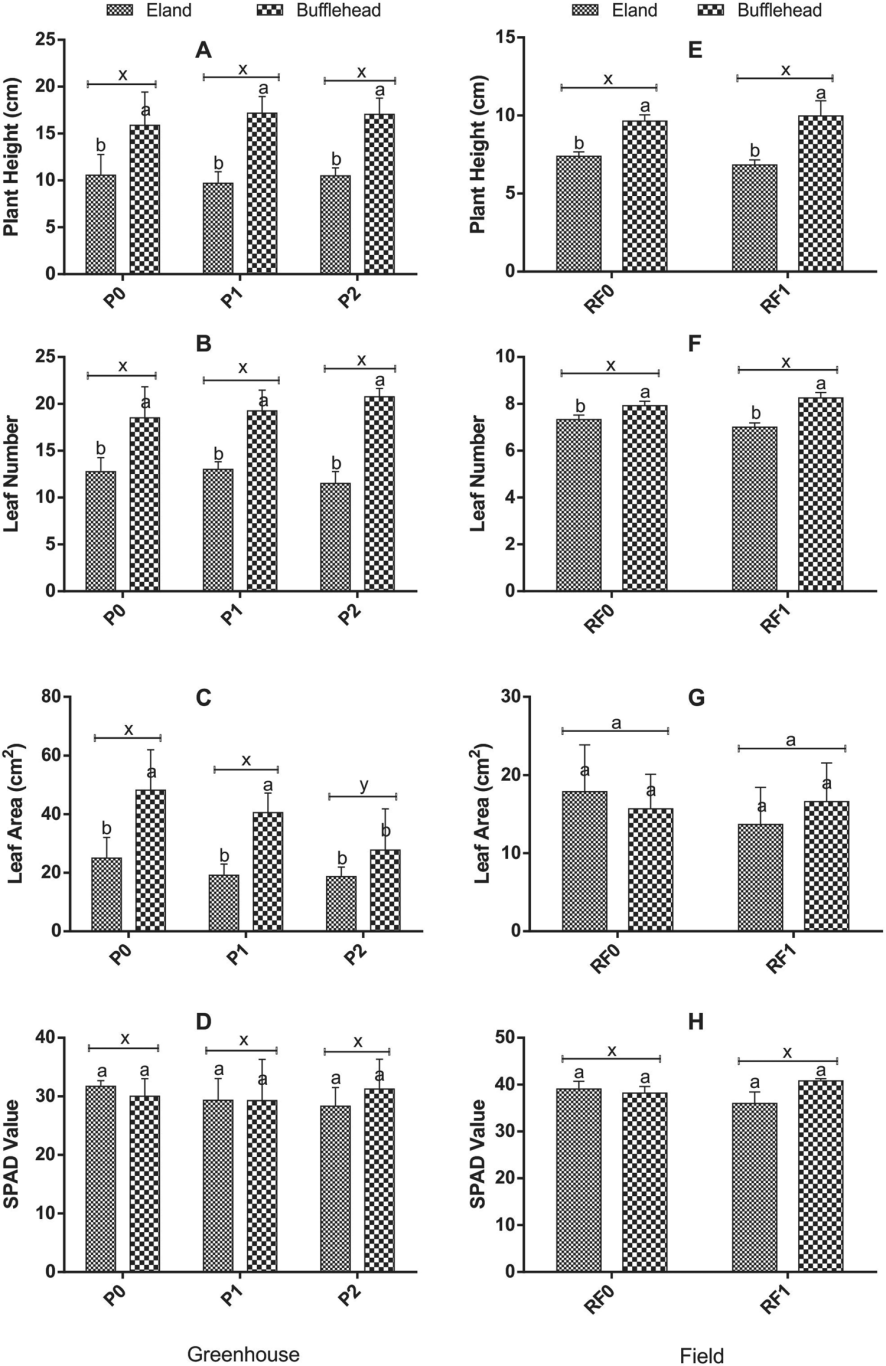
Effect of OPV-RF Transmittance, Genotype and Interaction on Growth and SPAD Value of Spinach in Greenhouse and Field Conditions. Bars show mean and standard deviations. Bars with different letters are significantly different at *p* < 0.05. P0: Plants grown under no OPV in greenhouse; P1: plants grown under OPV with transmittance peak of 0.64; P2: plants grown under OPV with transmittance peak of 0.11; RF0: plants grown under no red-foil in the field; RF1: plants grown under red-foil with transmittance peak of 0.89

The effects of OPV-RF transmittance on PH, LN, LA and SPAD value of spinach are shown in Fig. 3. OPV transmittance did not significantly affect (*p* > 0.05) PH, LN, and SPAD value of spinach under greenhouse conditions (Fig. 3A–D). Significant effect was recorded for LA with P0 (36.63 ± 16.04) showing comparable (*p* > 0.05) LA with P1 (29.29 ± 12.49) but was larger than P2 (*p* < 0.05) (Fig. 3C). In a similar pattern, there was no significant effect (*p* > 0.05) of RF transmittance on PH, LN, LA, and SPAD value of spinach in the field (Fig. 3E, F).

Bufflehead genotype performed significantly better than eland in PH, LN, and LA across both environments (Fig. 3). Plants grown in the greenhouse outperformed the plants raised under field conditions in all growth traits measured. The highest LN in the field was 8.25 compared to 19.50 in the greenhouse. The tallest plant on the field was about 10.05 cm high in contrast to 16.70 cm in the greenhouse. SPAD value did not differ between genotypes of spinach either in field or in the greenhouse.

Under controlled greenhouse conditions, the response of bufflehead and eland genotypes to OPV-transmittance were similar (*p* > 0.05) in PH and SPAD value but varied (*p* < 0.05) in LN and LA. Interactions of bufflehead and no-OPV (BP0), bufflehead and OPV1 (BP1) and bufflehead and OPV2 (BP2) were statistically similar but performed higher than interactions of eland with no-OPV (EP0), eland and OPV1 (EP1) and eland with OPV2 (EP2) which were also similar in LN. The interaction BP0 (48.25 ± 13.79) and BP1 (40.57 ± 6.65) produced the largest LA in contrast to the interaction EP2 (18.68 ± 3.31) which was the least. In like manner, the interaction effect of RF and genotype did not show significant difference for PH, LN, LA, and SPAD value of spinach under field conditions.

### Photo-irradiance responses of ETR, Y(II), Y(NPQ) and Y(NO) in OPV and control conditions

**Fig. 4:**
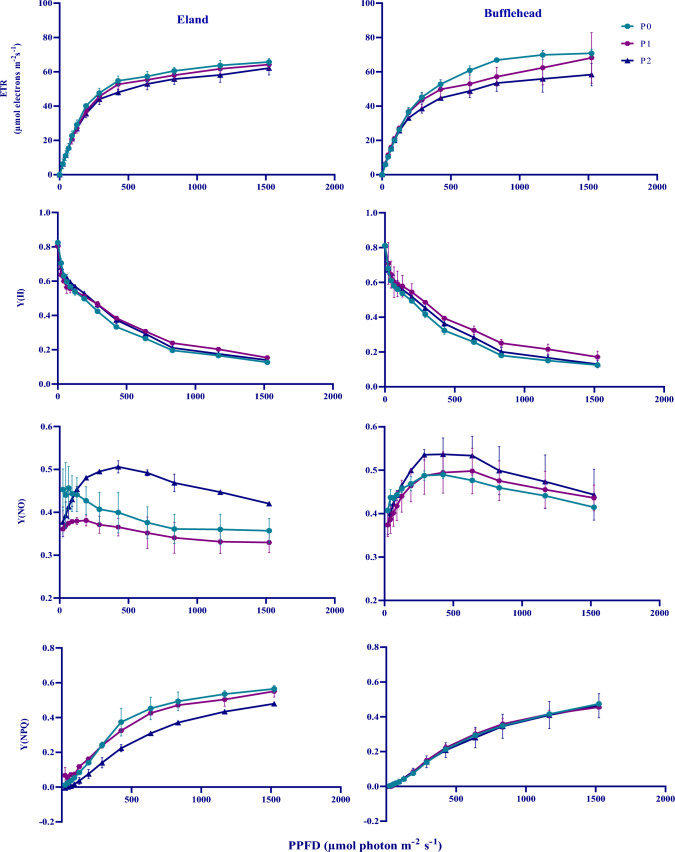
Photo-Irradiance response curves of ETR, Y(II), Y(NO) and Y(NPQ). ETR: electron transport rate; Y(II): effective photochemical yield; Y(NO): yield of non-photochemical energy losses via heat dissipation and fluorescence at the reaction centers; Y(NPQ): yield of non-photochemical energy losses via heat dissipation at the antenna. P0: control; P1: plants grown under OPV with transmittance peak of 0.64; P2: plants grown under OPV with transmittance peak of 0.11; Data are Mean ± SD (*n* = 3)

**Fig. 5:**
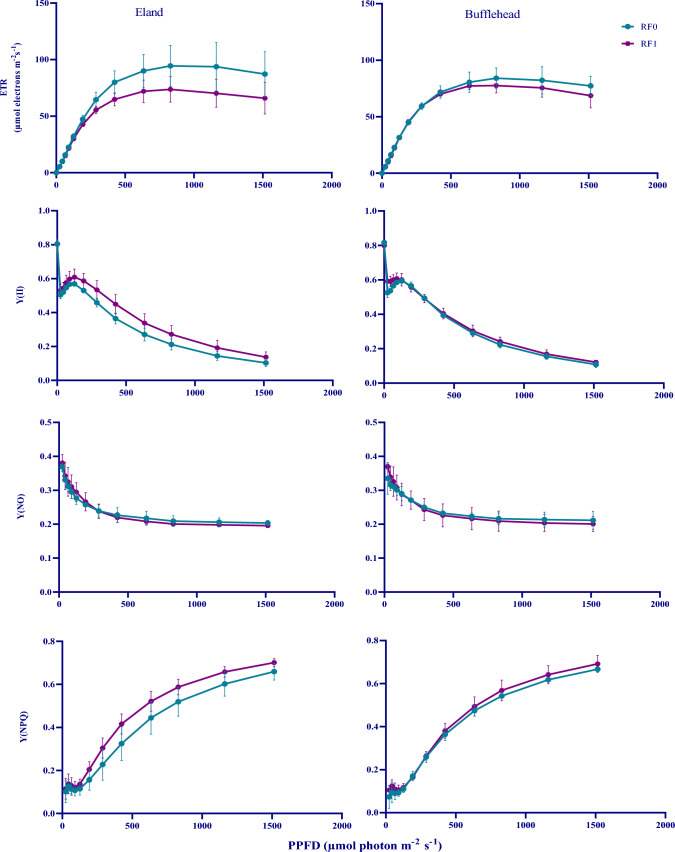
Photo-Irradiance response curves of ETR, Y(II), Y(NO) and Y(NPQ) of Spinach Plants Grown in Field. ETR: electron transport rate; Y(II): effective photochemical yield; Y(NO): yield of non-photochemical energy losses via heat dissipation and fluorescence at the reaction centers; Y(NPQ): yield of non-photochemical energy losses via heat dissipation at the antenna. RF0: control; RF1: plants grown under red-foil with transmittance peak of 0.89. Data are Mean ± SD (*n* = 3)

The photo-irradiance responses of ETR, Y(II), Y(NPQ) and Y(NO) are shown in Figs. 4 and 5 for greenhouse and field, respectively. ETR increased progressively with increasing light intensity across genotypes and environments until a saturation point is reached beyond which ETR started to decline. The response was similar in OPV, RF and control plants at low light intensities (< 400 PPFD). However, at higher light intensities (> 500 PPFD) variation became evident. OPV and RF plants generally showed reduced ETR at > 500 PPFD compared to control. Eland genotype was more sensitive to light at > 500 PPFD compared to bufflehead. OPV and RF plants generally showed higher photochemical efficiency than control plants across environments and genotypes. Y(II) declined progressively with increasing light intensity (> 200 PPFD). P2 plants showed higher values of Y(NO) with increasing light intensity across genotypes compared to P0 and P1 plants in the greenhouse (Fig. 4). However, there was no variation in Y(NO) across RF and genotypes in the field (Fig. 5).

### Effect of OPV-RF transmittance, genotype and interaction on photosynthesis of spinach

**Fig. 6:**
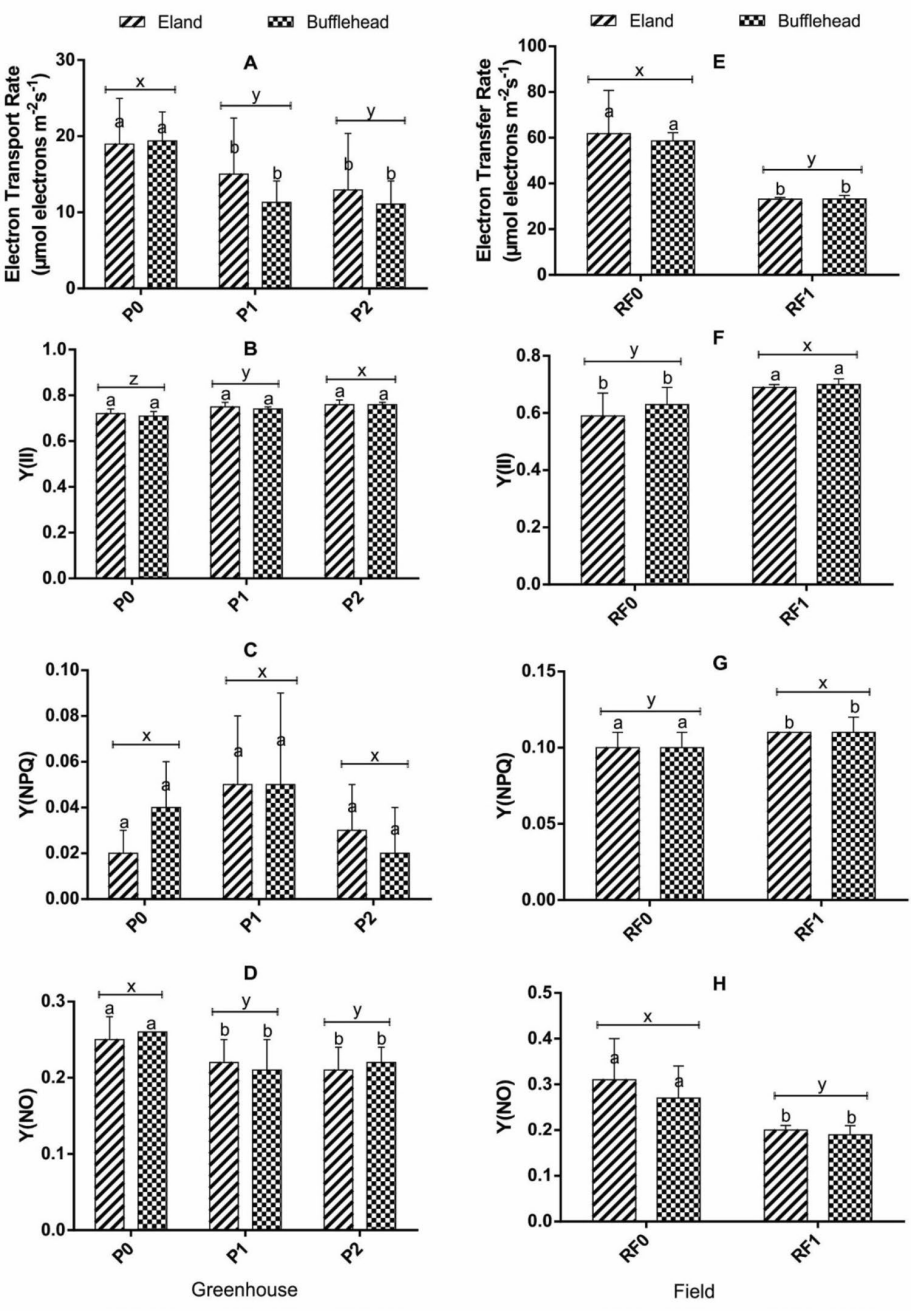
Effect of OPV-RF Transmittance, Genotype and Interaction on Photosynthesis of Spinach in Greenhouse and Field Conditions. Bars show mean and standard deviations. Bars with different letters are significantly different at *p* < 0.05. P0: Plants grown under no OPV in greenhouse; P1: plants grown under OPV with transmittance peak of 0.64; P2: plants grown under OPV with transmittance peak of 0.11; RF0: plants grown under no red-foil in the field; RF1: plants grown under red-foil with transmittance peak of 0.89; Y(II): effective photochemical yield; Y(NO): yield of non-photochemical energy losses via heat dissipation and fluorescence at the reaction centers; Y(NPQ): yield of non-photochemical energy losses via heat dissipation at the antenna

The effects of OPV-RF transmittance on photosynthetic parameters of spinach are shown in Fig. 6. P1 and P2 significantly reduced ETR and Y(NPQ) compared to P0 which recorded higher values. In addition, P2 (0.76 ± 0.01) and P1 (0.74 ± 0.01) had higher Y(II) than P0 (0.72 ± 0.02) (Fig. 6B). Y(NO) was not affected by OPV transmittance in the greenhouse. Likewise, RF significantly reduced ETR, Y(NO) and Y(NPQ) in the field while Y(II) was significantly improved (Fig. 6E–H). There was no significant variation in the two genotypes for all traits measured both in greenhouse and field conditions (Fig. 6). The response of the two genotypes to different light transmittance in the greenhouse and in field were similar (*p* > 0.05) for all photosynthetic traits measured across the duration of the study.

## Discussion

### Low OPV transmittance reduced yield attributes of spinach in greenhouse

The transmittance properties of the materials used over a plant can influence physiological processes, and consequently growth and yield attributes of the plant. It is a very important parameter because; it regulates the amount and quality of light absorbed by leaves. It also provides a basis to explain variation in photosynthetic efficiencies as a function of different light spectra it transmits. According to Liu and Van lersel (2021) light intensity and quality can affect physiological and yield processes. In this study, P1 with a higher transmittance of red light (RL) (64%) and blue light (BL) (11%) was statistically similar (*p* > 0.05) to P0 in FSW, DSW, TBW and was superior to P0 in TED. However, P2 with lower transmittance of RL (11%) and BL (9%) influenced lower values in all the yield traits (Fig. 2). The higher root weight of P0 plants (0.15) in contrast to the higher TED of P1 (15.27) and P2 (10.99) is an indication that OPV plants utilized greater proportion of the absorbed light energy for shoot production as against root development. LMA was not significantly different among treatments although P1 and P2 had insignificantly higher value (0.02) compared to P0 (0.01) implying that the plants did not sufficiently adjust their leaves to reduced light. The reduced yield and yield traits as a consequence of reduced light observed in this study corroborates previous reports on other crops (Islam et al. 1993; Mu et al. 2010; Wang et al. 2015; Schmierer et al. 2021).

There was no significant difference in plant height, and leaf number which is consistent with the report of Chidburee et al. (2017) in *Curcuma alismatifolia* grown under RL condition. P2 plants recorded higher LA than P1 and P0 by maintaining higher photochemical efficiency and the least non-photochemical energy losses to heat and fluorescence at the reaction centers (Fig. 6). Reduced LA as a function of reduced light was reported by Schmierer et al. (2021) on the contrary. The leaf is an indispensable part of a plant where virtually all photosynthetic activities take place, and for spinach, it is also the economic part of the plant. Plants are able to harvest light energy from the sun by virtue of its antenna contained in the chloroplast of leaves (Lambers et al. 2008), and could adjust their antenna system to suit the prevailing environmental condition through leaf and chlorophyll movements (Muller et al. 2001). For instance, at low light intensity, plants tend to increase the size of their antenna and reduce it at very high light intensity (Fryer et al. 1998) as a protective mechanism to regulate the quantum of light energy that is absorbed and transmitted to the reaction centers, which explains why P2 with the least transmittance recorded the largest LA.

### RF transmittance reduced yield attributes of spinach under extreme cold conditions in the field

RF transmitted majorly monochromatic RL (89%) with very infinitesimal proportion of BL (< 1%), which impacted on shoot weight, total biomass weight and photosynthesis of spinach in extreme field condition. RF in contrast to full irradiance significantly reduced FSW, DSW, TBW, LMA and TED under field conditions. The reduction in shoot weight, biomass, leaf mass per area and total energy distribution of spinach across genotypes is suggestive of the RF’s inability to supply complementary blue light spectra which is a major contributor to photosynthesis as it has been reported to influence leaf expansion, stomata increase and opening as well as maximum and effective quantum yields of photosystem II photochemistry (Miao et al. 2015) which directly impacts crop yield. This report is in tandem with earlier reports of Matsuda et al. (2008) and Hogewoning et al. (2010) who asserted that a combination of BL and RL could promote plant biomass, photosynthesis and chlorophyll concentration of plants under similar light intensity. PH, LN, and LA were unaffected by RF transmittance. Plants under RF1 were unable to adjust their leaves due to the quality and intensity of light transmitted (Liu and Van lersel 2021) in addition to extreme cold weather. Similarity in growth pattern of plant under RL to control was previously corroborated by other scientists (Chidburee et al. 2017; Ezzaeri et al. 2018; Anusiya and Sivachandiran 2019; Liu et al. 2019; Hassanien et al. 2022).

### SPAD value was unaffected by OPV-RF transmittance in greenhouse and field conditions

OPV and RF transmittance did not significantly affect SPAD value of spinach across environments which is consistent with the findings of Yang et al. (2019) who reported non-significant effect of shading on chlorophyll *a* and *b* content of three forage species; Schmierer et al. (2021) who reported comparable chlorophyll *a* and *b* contents of super dwarf rice for three shading levels in the greenhouse; Hassanien et al. (2022) who found non-significant increase in SPAD value of shaded chili pepper compared to control plants. Chlorophyll content of a leaf could be a very good determinant of photosynthetic efficiency and chloroplast development as they influence the ability of plants to harvest solar radiation for photosynthesis. The similarity in SPAD values of shaded and control plants could be an advantage to an APV farmer in that, they can produce crops and electricity from a limited space without affecting the chlorophyll content of the crop negatively. The non-significant increase in chlorophyll content of plants grown under OPV and RF is an acclimation to insufficient light which could be beneficial since the nutritional value of leafy vegetables tend to increase with larger chlorophyll content.

### Genotype, genotype-OPV interaction and genotype-RF interaction affected growth and biomass of spinach

The genotypes expressed clear genotypic variation in growth and yield attributes at both environments. Bufflehead influenced significantly higher FSW, DSW, TBW, PH, LN and LA than eland genotype across both environments. Genotypes responded differently to OPV-transmittance in the greenhouse. Interaction of BP0 and BP1 were comparable in FSW, DSW, TBW, LMA and TED but were superior to BP2, EP0, EP1 and EP2 interactions, respectively. With an exception of LN where interaction of BRF1 and BRF0 recorded higher values than ERF0 and ERF1, all other measured traits did not significantly differ across all interacting factors in the field. The variation in growth and yield attributes of spinach as a consequence of genotype across both environments could be implicated on their preference for shade environments (Jiwuba et al. 2020). Bufflehead genotype which is shade loving showed statistical similarity for most growth and yield indices when grown under full irradiance and when grown under P1 which could suggest that P1 transmitted sufficient irradiance necessary for normal photosynthesis and other physiological processes of the plant in contrast to P2 which significantly reduced growth and yield attributes across genotypes. Conversely, Eland genotype which is shade shy showed reduced growth and yield attributes under shade. This report is consistent with Malaviya et al. (2020) who reported differential responses of *Megathyrsus maximus* genotypes to shading intensity.

### Effect of OPV-RF transmittance on photosynthesis of spinach

The lower CO_2_ assimilation rates of P1, P2 and RF1 plants were due to reduced light effect of the materials. OPV and RF transmittance generally influenced higher Y(II) values in both environments. However ETR, Y(NPQ) and Y(NO) were lower than control. The higher photochemical efficiencies of OPV and RF treated plants and the lower non photochemical energy losses at the antenna Y(NPQ) and reaction centers Y(NO) could be implicated on the effect of the materials in reducing the incident radiation reaching the plants thereby minimizing the potential for photo-oxidative damage. P0 and RF0 plants were unable to effectively convert greater proportion of their excitation energy to photochemistry due to higher non-photochemical energy losses via the regulated Y(NPQ) and unregulated (Y(NO) pathways. Increased photosynthetic efficiencies and decreased non photochemical quenching in reduced light have been reported in other crop species (Gong et al. 2015; Wang et al. 2016; Song and Li 2016; Ravishankar et al. 2021; Schmierer et al. 2021). In addition, by reducing the incident radiation, the amount of absorbed light reaching the reaction centers was greatly reduced. The low Y(NO) values recorded relative to Y(NPQ) across all levels suggest that both photochemical energy conversion and protective regulatory mechanisms are efficient and that the plants efficiently managed the incident radiation (Busch et al. 2009).

### Effect of OPV-RF transmittance on photo-irradiance response curves

The photo-irradiance response curves followed a similar trend in both OPV and RF plants. At increasing light intensities (> 400 PPFD) after saturation, CO_2_ assimilation rate was higher in P0 than P1 and P2. At similar light intensities, P1 and P2 converted higher proportion of their excitation energy to photochemistry as against P0 where substantial proportion was dissipated as heat at the antenna via the Y(NPQ) pathway (Fig. 2). P2 plants expressed the highest non photochemical energy losses at the reaction centers via the Y(NO) pathway and the least regulated energy losses to heat via the Y(NPQ) pathway, an expression of its inability to protect itself from excess light as a consequence of been grown under reduced light intensity. In contrast, P1 had low non photochemical energy losses at the reaction centers and comparable photo protective ability to P0 across both genotypes due to their prior acclimation to higher incident radiation in the growth environment which is in agreement with Ravishankar et al. (2021) and Schmierer et al. (2021) who reported higher photochemical efficiency, higher non-regulated energy losses to heat and fluorescence, and lower regulated non-photochemical energy losses to heat in plants acclimated to reduced light prior to commencement of rapid light curves.

For field plants, RF1 did not vary much from RF0 (Fig. 3). With the exception of CO_2_ assimilation rate which was slightly higher in RF0, effective photochemical yield, yield of non-photochemical energy losses to heat and fluorescence at the reaction centers, and yield of regulated non-photochemical energy losses at the antenna were comparable in bufflehead genotype. Variation however, was more pronounced in eland genotype. In general, plants grown under high OPV or RF transmittance handled excess light more efficiently than those grown under low OPV transmittance. This finding corroborates Schmierer et al. (2021) who reported that on exposure to strong light, plants grown under the highest light intensities handled excessive light energy more efficiently.

## Conclusions

The transmittance properties of PV materials installed over plants in an APV system is fundamental as it influences both light quality and intensity. Light quality and intensity can affect physiological and yield processes of plants. In this study, we confirmed significant reduction in shoot weight and total biomass of spinach grown under very low light intensities as a function of the transmittance properties of the OPV cell used (P2). Interestingly, P1 with high transmittance properties and good balance of blue and red light was comparable to control in most of the growth and yield traits measured which confirms our hypothesis. In addition, it influenced better shoot to root distribution than control. In general, greenhouse plants outperformed field plants due to the improved micro-climate environment of the greenhouse relative to the field.

Light quality in terms of spectra or colors of light that a plant is exposed to can also influence performance. This was confirmed in this study as RF reduced shoot and total biomass production of spinach in the field and could be implicated on its inability to transmit other complementary light spectra aside RL. OPV-RF transmittance did not affect plant height (PH), leaf number (LN), and SPAD value but leaf area (LA) was highest in P2. Photochemical energy conversion was higher in P1, P2 and RF1 in contrast to control due to lower levels of non-photochemical energy losses through the Y(NO) and Y(NPQ) pathways.

Photo-irradiance curves showed that plants grown under reduced light (P2) did not efficiently manage excess light when exposed to high light intensities. Bufflehead genotype showed superior growth and yield traits than eland across OPV-RF levels.

Conclusively, agrivoltaics is an emerging technology that could potentially provide the key to the food and energy challenges in the tropics without compromising crop yield. This study is also a confirmation that photovoltaics can successfully be integrated with crop production without significant reduction in yield traits. P1 installed over spinach plants in the greenhouse competed favorably with the control plants in fresh and dry shoot weight, fresh and dry biomass weight and leaf mass per area. However, decrease in shoot and biomass yield was observed with RF1 plants in the field, and also with P2 plants in the greenhouse principally as a result of insufficient light. It is therefore important to strike the right balance in terms of using the right PV materials, transmittance, distance from the plant, installation angle etc., to resolve some of the challenges at least in the short term. Perhaps with further experimentations, the ideal mix with respect to the panel type, transmittance, installation angle and height for enhanced efficiency of the system will be obtained. On the basis of these findings, it is recommended that OPV cells with transmittance properties greater than or equal to 11% in BL and 64% in RL be used in APV systems for improved efficiency.

## Supplementary Information

Below is the link to the electronic supplementary material.

Supplementary file1 (DOCX 29 KB)

## Data Availability

All data generated or analyzed during this study can be requested from the corresponding author.

## Abbreviations

APV: Agrophotovoltaics
BP0: Interaction of B and P0
BP1: Interaction of B and P1
BP2: Interaction of B and P2
BRF0: Interaction of B and RF0
BRF1: Interaction of B and RF1
DRW: Dry root weight
DSW: Dry shoot weight
EP0: Interaction of E and P0
EP1: Interaction of E and P1
EP2: Interaction of E and P2
ERF0: Interaction of E and RF0
ERF1: Interaction of E and RF1
ETR: Electron transport rate
FSW: Fresh shoot weight
LA: Leaf area
LMA: Leaf mass per area
LN: Leaf number
OPV: Organic photovoltaics
P0: Plants growing under no OPV
P1: Plants growing under OPV with transmittance peak of 0.64
P2: Plants growing under OPV with transmittance peak of 0.11
PH: Plant height
PPFD: Photosynthetic photon flux density
RF: Red-foil
RF0: Plants growing under no red-foil
RF1: Plants growing under red-foil with transmittance peak of 0.89
TBW: Total biomass weight
TED: Total energy distribution
WAIOPV: Weeks after installation of OPV
WAIRF: Weeks after installation of RF
Y(II): Effective photochemical yield
Y(NO): Yield of non-photochemical energy losses to heat and fluorescence at the reaction centers
Y(NPQ): Yield of non-photochemical energy losses to heat at the antenna
