# Supplementary material for: Effect of organic photovoltaic and red-foil transmittance on yield, growth and photosynthesis of two spinach genotypes under field and greenhouse conditions
Source: Photosynth Res. 2023 Jun 14;157(2-3):103–18. doi: 10.1007/s11120-023-01028-8 (PMC10485117; doi:10.1007/s11120-023-01028-8)
Supplement: Supplementary file 1 — Supplementary file1 (DOCX 29 KB) [file 11120_2023_1028_MOESM1_ESM.docx]

| **Table 1a: Effect of OPV Transmittance and Genotype on Leaf Yield and Yield Traits of Spinach in the Greenhouse** | | | | | | | |
| --- | --- | --- | --- | --- | --- | --- | --- |
| Treatment | FSW (g) | DSW (g) | DRW (g) | TBW (g) | LMA (g cm^-3^) | | TED |
| *Shading Effect* | | | | | | | |
| P0 | 9.60±5.13a | 0.40±0.23ab | 0.15±0.16a | 0.55±0.21a | | 0.01±0.00a | 5.79±4.25c |
| P1 | 8.69±5.18a | 0.49±0.26a | 0.03±0.02b | 0.52±0.28a | | 0.02±0.00a | 15.27±2.29a |
| P2 | 5.53±2.00b | 0.35±0.17b | 0.03±0.01b | 0.38±0.18b | | 0.02±0.01a | 10.99±3.62b |
| p-value | 0.03 | 0.05 | 0.02 | 0.06 | | 0.11 | 0.00 |
| LSD | 3.03 | 0.11 | 0.08 | 0.15 | | NS | 3.71 |
|  |  |  |  |  | |  |  |
| *Genotype Effect* | | | | | | | |
| E | 4.91±1.67b | 0.23±0.03b | 0.09±0.15a | 0.32±0.15b | | 0.02±0.00a | 9.23±6.43a |
| B | 10.98±4.48a | 0.59±0.16a | 0.05±0.01a | 0.65±0.17a | | 0.01±0.01b | 12.03±3.96a |
| p-value | 0.00 | 0.00 | 0.25 | 0.00 | | 0.01 | 0.06 |
| LSD | 2.49 | 0.09 | NS | 0.12 | | 0.00 | NS |
|  |  |  |  |  | |  |  |
|  |  |  |  |  | |  |  |
| *Interaction Effect* | | | | | | | |
| EP0 | 6.05±2.25b | 0.22±0.18c | 0.23±0.19a | 0.45±0.20bc | | 0.01±0.00a | 2.41±3.07a |
| EP1 | 4.68±1.37b | 0.26±0.08c | 0.17±0.00a | 0.28±0.01c | | 0.02±0.00a | 15.77±2.29a |
| EP2 | 4.00±0.59b | 0.20±0.02c | 0.22±0.01a | 0.22±0.02c | | 0.02±0.00a | 10.00±5.03a |
| BP0 | 13.15±4.76a | 0.58±0.18ab | 0.06±0.01b | 0.64±0.20ab | | 0.01±0.00a | 9.17±1.49a |
| BP1 | 12.70±4.22a | 0.71±0.16a | 0.05±0.01b | 0.76±0.16a | | 0.01±0.01a | 15.01±1.64a |
| BP2 | 7.08±1.62b | 0.50±0.81b | 0.04±0.01b | 0.55±0.09b | | 0.01±0.01a | 11.97±1.64a |
| p-value | 0.00 | 0.00 | 0.04 | 0.00 | | 0.37 | 0.07 |
| LSD | 4.31 | 0.16 | 0.12 | 0.21 | | NS | NS |
| FSW: fresh shoot weight; DSW: dry shoot weight; DRW: dry root weight; TBW: total biomass weight; LMA: leaf mass per area; TED: total energy distribution; P0: control; P1: plants grown under photovoltaic panel with transmittance peak of 0.64; P2: plants grown under photovoltaic panel with transmittance peak of 0.11; B: bufflehead; E: eland; EP0: Interaction of E and P0; EP1: interaction of E and P1; EP2: interaction of E and P2; BP0: interaction of B and P0; BP1: interaction of B and P1; BP2: interaction of B and P2; NS: non-significant; LSD: least significant difference for comparing two treatment means. Values shown are means with standard deviations. Means with different alphabets are significantly different at p<0.05 for each corresponding treatment effect. | | | | | | | |

| **Table 1b: Effect of RF Transmittance and Genotype on Leaf Yield and Yield Traits of Spinach in the Field** | | | | | | |
| --- | --- | --- | --- | --- | --- | --- |
| Treatment | FSW | DSW | DRW | TBW | LMA | TED |
| *Shading Effect* | | | | | | |
| RF0 | 2.99±0.66a | 0.42±0.05a | 0.03±0.01a | 0.45±0.03a | 0.03±0.00a | 15.90±10.02a |
| RF1 | 2.13±0.50b | 0.28±0.08b | 0.04±0.01a | 0.31±0.08b | 0.02±0.01b | 7.60±2.33b |
| p-value | 0.00 | 0.00 | 0.43 | 0.00 | 0.03 | 0.02 |
| LSD | 0.55 | 0.05 | NS | 0.05 | 0.00 | 7.80 |
|  |  |  |  |  |  |  |
| *Genotype Effect* | | | | | | |
| E | 2.22±0.01b | 0.309±0.10b | 0.04±0.01a | 0.35±0.10b | 0.03±0.01a | 8.10±2.41b |
| B | 2.89±0.70a | 0.387±0.09a | 0.03±0.01a | 0.42±0.08a | 0.02±0.01a | 15.40±10.45a |
| p-value | 0.02 | 0.00 | 0.21 | 0.00 | 0.23 | 0.04 |
| LSD | 0.55 | 0.05 | NS | 0.05 | NS | 7.80 |
|  |  |  |  |  |  |  |
| *Interaction Effect* | | | | | | |
| ERF0 | 2.63±0.58a | 0.40±0.01a | 0.04±0.00a | 0.43±0.13a | 0.03±0.01a | 10.20±0.24a |
| ERF1 | 1.81±0.17a | 0.22±0.01a | 0.04±0.01a | 0.26±0.24a | 0.02±0.01a | 6.00±1.54a |
| BRF0 | 3.34±0.60a | 0.45±0.60a | 0.03±0.02a | 0.47±0.42a | 0.02±0.00a | 21.7±12.29a |
| BRF1 | 2.44±0.50a | 0.33±0.83a | 0.04±0.00a | 0.36±0.85a | 0.02±0.01a | 9.20±1.95a |
| p-value | 0.99 | 0.07 | 0.67 | 0.07 | 0.34 | 0.38 |
| LSD | NS | NS | NS | NS | NS | NS |
| FSW: fresh shoot weight; DSW: dry shoot weight; DRW: dry root weight; TBW: total biomass weight; LMA: leaf mass per area; TED: total energy distribution; RF0: control; RF1: plants grown under red-foil with transmittance peak of 0.89; B: bufflehead; E: eland; ERF0: Interaction of E and RF0; ERF1: interaction of E and RF1; BRF0: interaction of B and RF0; BRF1: interaction of B and RF1; NS: non-significant; LSD: least significant difference for comparing two treatment means. Values shown are means with standard deviations. Means with different alphabets are significantly different at p<0.05 for each corresponding treatment effect. | | | | | | |

| **Table 2a: Effect of OPV Transmittance and Genotype on Growth and SPAD Value of Spinach in Greenhouse** | | | | | | | | |
| --- | --- | --- | --- | --- | --- | --- | --- | --- |
|  |  |  |  |  | |  |  |  |
| Treatment | Plant Height (cm) | |  | Leaf Number | | | LA (cm^2^) | SPAD Value |
|  | 3 WAIOPV | 4 WAIOPV |  | 3 WAIOPV | 4 WAIOPV | |  |  |
| *Shading Effect* | | | | | | | | |
| P0 | 11.94±4.17a | 13.21±3.95a |  | 14.75±2.75a | 15.62±3.89a | | 36.63±16.04a | 30.86±2.26a |
| P1 | 11.80±3.03a | 13.44±4.24a |  | 15.25±0.96a | 16.12±3.68a | | 29.90±12.49a | 29.29±3.73a |
| P2 | 12.45±3.21a | 13.78±3.74a |  | 15.50±1.29a | 16.12±5.05a | | 23.24±10.62b | 29.79±3.20a |
| p-value | 0.83 | 0.87 |  | 0.85 | 0.83 | | 0.03 | 0.76 |
| LSD | NS | NS |  | NS | NS | | 9.65 | NS |
|  | | | | | | | | |
| *Genotype Effect* | | | | | | | | |
| E | 10.24±1.46b | 10.92±0.52b |  | 9.46±1.40b | 12.42±1.31b | | 20.95±5.45b | 29.78±3.01a |
| B | 16.70±2.34a | 15.17±1.70a |  | 14.67±2.62a | 19.50±2.36a | | 38.88±13.98a | 30.18±4.89a |
| p-value | 0.00 | 0.00 |  | 0.00 | 0.00 | | 0.00 | 0.82 |
| LSD | 1.94 | 1.78 |  | 1.15 | 1.62 | | 7.88 | NS |
|  | | | | | | | | |
| *Interaction Effect* | | | | | | | | |
| EP0 | 9.00±0.98a | 10.55±2.22a |  | 11.00±0.00b | 12.75±1.50b | | 25.02±7.08c | 31.70±0.99a |
| EP1 | 9.75±2.11a | 9.70±1.23a |  | 11.25±0.50b | 13.00±0.82b | | 19.16±3.80c | 29.33±3.73a |
| EP2 | 9.63±1.15a | 10.48±0.86a |  | 10.50±0.58b | 11.50±1.29b | | 18.68±3.31c | 28.33±3.20a |
| BP0 | 14.88±4.06a | 15.88±3.54a |  | 14.75±2.75a | 18.50±3.32a | | 48.25±13.79a | 30.02±3.00a |
| BP1 | 13.85±2.41a | 17.18±1.77a |  | 15.25±0.96a | 19.25±2.22a | | 40.57±6.65ab | 29.25±7.07a |
| BP2 | 15.27±1.23a | 17.05±1.73a |  | 15.50±1.29a | 20.75±0.96a | | 27.80±14.02bc | 31.25±5.09a |
| p-value | 0.69 | 0.59 |  | 0.00 | 0.00 | | 0.00 | 0.85 |
| LSD | NS | NS |  | 1.99 | 2.81 | | 13.65 | NS |
| WAIOPV: weeks after installation of photovoltaic panels (PV installation date: 10.01.2022); P0: control; P1: photovoltaic panel with transmittance peak of 0.64; P2: photovoltaic panel with transmittance peak of 0.11; B: bufflehead; E: eland; EP0: Interaction of E and P0; EP1: interaction of E and P1; EP2: interaction of E and P2; BP0: interaction of B and P0; BP1: interaction of B and P1; BP2: interaction of B and P2; NS: non-significant; LA: leaf area; LSD: least significant difference for comparing two treatment means. Values shown are means with standard deviations. Means with different alphabets are significantly different at p<0.05 for each corresponding treatment effect. | | | | | | | | |

| Table 3a: Effect of OPV-Transmittance and Genotype on Photosynthesis of Spinach in Controlled Greenhouse Environment | | | | | | | | | | | | |
| --- | --- | --- | --- | --- | --- | --- | --- | --- | --- | --- | --- | --- |
| Treatment | ETR | |  | Y(II) | | |  | Y(NO) | |  | Y(NPQ) | |
|  | 6 WAIOPV | 7 WAIOPV |  | 6 WAIOPV | 7 WAIOPV | |  | 6 WAIOPV | 7 WAIOPV |  | 6 WAIOPV | 7 WAIOPV |
| *Shading Effect* | | | | | | | | | | | | |
| P0 | 20.53±4.20a | 19.18±4.70a |  | 0.72±0.02c | 0.72±0.02c |  | | 0.05±0.02a | 0.03±0.02a |  | 0.24±0.01a | 0.25±0.02a |
| P1 | 11.86±1.60b | 13.45±5.60b |  | 0.74±0.01b | 0.74±0.01b |  | | 0.04±0.02a | 0.05±0.03a |  | 0.22±0.03b | 0.22±0.03b |
| P2 | 10.66±1.20b | 12.01±5.30b |  | 0.76±0.01a | 0.76±0.01a |  | | 0.04±0.02a | 0.03±0.02a |  | 0.20±0.03c | 0.22±0.02b |
| p-value | 0.00 | 0.04 |  | 0.00 | 0.00 |  | | 0.62 | 0.21 |  | 0.00 | 0.02 |
| LSD | 2.93 | 5.68 |  | 0.02 | 0.02 |  | | NS | NS |  | 0.02 | 0.03 |
|  |  |  |  |  |  |  | |  |  |  |  |  |
| *Genotype Effect* | | | | | | | | | | | | |
| E | 14.84±5.80a | 15.83±6.80a |  | 0.74±0.02a | 0.74±0.02a |  | | 0.05±0.02a | 0.03±0.02a |  | 0.21±0.03a | 0.23±0.03a |
| B | 13.87±4.70a | 13.93±4.90a |  | 0.74±0.03a | 0.73±0.02a |  | | 0.04±0.02a | 0.04±0.03a |  | 0.23±0.02a | 0.23±0.03a |
| p-value | 0.41 | 0.39 |  | 0.47 | 0.18 |  | | 0.58 | 0.70 |  | 0.10 | 0.63 |
| LSD | NS | NS |  | NS | NS |  | | NS | NS |  | NS | NS |
|  |  |  |  |  |  |  | |  |  |  |  |  |
| *Interaction Effect* | | | | | | | | | | | | |
| EP0 | 21.68±4.80a | 18.95±6.00a |  | 0.72±0.02a | 0.73±0.02a |  | | 0.04±0.02a | 0.02±0.01a |  | 0.24±0.01a | 0.25±0.03a |
| EP1 | 11.81±1.80a | 15.00±7.40a |  | 0.75±0.01a | 0.73±0.02a |  | | 0.05±0.03a | 0.05±0.03a |  | 0.20±0.02a | 0.22±0.03a |
| EP2 | 11.01±1.60a | 12.95±7.40a |  | 0.76±0.02a | 0.76±0.02a |  | | 0.05±0.02a | 0.03±0.02a |  | 0.20±0.03a | 0.21±0.03a |
| BP0 | 19.38±3.80a | 19.40±3.80a |  | 0.71±0.02a | 0.71±0.02a |  | | 0.06±0.02a | 0.04±0.02a |  | 0.23±0.00a | 0.26±0.00a |
| BP1 | 11.90±1.70a | 11.30±2.80a |  | 0.74±0.01a | 0.74±0.01a |  | | 0.03±0.01a | 0.05±0.04a |  | 0.23±0.04a | 0.21±0.04a |
| BP2 | 10.32±0.80a | 11.08±3.02a |  | 0.76±0.02a | 0.75±0.01a |  | | 0.03±0.02a | 0.02±0.02a |  | 0.22±0.02a | 0.22±0.02a |
| p-value | 0.69 | 0.69 |  | 0.62 | 0.18 |  | | 0.08 | 0.37 |  | 0.17 | 0.73 |
| LSD | NS | NS |  | NS | NS |  | | NS | NS |  | NS | NS |
| ETR: electron transport rate; Y(II): effective photochemical yield; Y(NO): yield of non-photochemical energy losses via heat dissipation and fluorescence at the reaction centers; Y(NPQ): yield of non-photochemical energy losses via heat dissipation at the antenna. WAIOPV: weeks after installation of photovoltaic panels (PV installation date: 10.01.2022); P0: control; P1: plants grown under photovoltaic panel with transmittance peak of 0.64; P2: plants grown under photovoltaic panel with transmittance peak of 0.11; B: bufflehead; E: eland; EP0: Interaction of E and P0; EP1: interaction of E and P1; EP2: interaction of E and P2; BP0: interaction of B and P0; BP1: interaction of B and P1; BP2: interaction of B and P2; NS: non-significant; LSD: least significant difference for comparing two treatment means. Values shown are means with standard deviations. Means with different alphabets are significantly different at p<0.05 for each corresponding treatment effect. | | | | | | | | | | | | |
|  | | | | | | | | | | | | |

| Table 3b: Effect of RF-Transmittance and Genotype on Photosynthesis of Spinach under Field Conditions | | | | | | | | | | | |
| --- | --- | --- | --- | --- | --- | --- | --- | --- | --- | --- | --- |
| Treatment | ETR | |  | Y(II) | |  | Y(NO) | |  | Y(NPQ) | |
|  | 6 WAIRF | 7WAIRF |  | 6 WAIRF | 7WAIRF |  | 6 WAIRF | 7WAIRF |  | 6 WAIRF | 7WAIRF |
| *Shading Effect* | | | | | | | | | | | |
| RF0 | 34.00±6.91a | 60.21±12.70a |  | 0.54±0.06b | 0.61±0.07b |  | 0.10±0.04a | 0.11±0.00a |  | 0.36±0.05a | 0.29±0.08a |
| RF1 | 20.18±2.80b | 33.57±1.23b |  | 0.63±0.03a | 0.70±0.02a |  | 0.09±0.01b | 0.10±0.01b |  | 0.26±0.04b | 0.19±0.02b |
| p-value | 0.00 | 0.00 |  | 0.00 | 0.00 |  | 0.00 | 0.00 |  | 0.00 | 0.00 |
| LSD | 5.95 | 11.80 |  | 0.05 | 0.06 |  | 0.00 | 0.00 |  | 0.06 | 0.06 |
|  |  |  |  |  |  |  |  |  |  |  |  |
| *Genotype Effect* | | | | | | | | | | | |
| E | 26.80±7.01a | 49.83±16.61a |  | 0.57±0.06a | 0.63±0.08a |  | 0.10±0.01a | 0.10±0.01a |  | 0.33±0.06a | 0.26±0.09a |
| B | 29.34±10.92a | 47.74±13.92a |  | 0.59±0.07a | 0.66±0.08a |  | 0.10±0.01a | 0.11±0.01a |  | 0.31±0.08a | 0.23±0.07a |
| p-value | 0.55 | 0.72 |  | 0.51 | 0.36 |  | 0.51 | 0.37 |  | 0.51 | 0.37 |
| LSD | NS | NS |  | NS | NS |  | NS | NS |  | NS | NS |
|  |  |  |  |  |  |  |  |  |  |  |  |
| *Interaction Effect* | | | | | | | | | | | |
| ERF0 | 31.03±6.10a | 61.78±18.92a |  | 0.53±0.04a | 0.59±0.08a |  | 0.09±0.01a | 0.10±0.01a |  | 0.37±0.05a | 0.31±0.09a |
| ERF1 | 21.17±2.82a | 33.91±0.84a |  | 0.63±0.03a | 0.69±0.01a |  | 0.10±0.00a | 0.11±0.00a |  | 0.27±0.02a | 0.20±0.01a |
| BRF0 | 36.97±7.04a | 58.63±3.61a |  | 0.55±0.06a | 0.63±0.06a |  | 0.09±0.01a | 0.10±0.01a |  | 0.36±0.06a | 0.27±0.07a |
| BRF1 | 19.19±2.90a | 33.23±1.60a |  | 0.64±0.04a | 0.70±0.02a |  | 0.11±0.01a | 0.11±0.00a |  | 0.25±0.05a | 0.19±0.02a |
| p-value | 0.14 | 0.82 |  | 0.95 | 0.67 |  | 0.95 | 0.67 |  | 0.95 | 0.67 |
| LSD | NS | NS |  | NS | NS |  | NS | NS |  | NS | NS |
| ETR: electron transport rate; Y(II): effective photochemical yield; Y(NO): yield of non-photochemical energy losses via heat dissipation and fluorescence at the reaction centers; Y(NPQ): yield of non-photochemical energy losses via heat dissipation at the antenna. WAIRF: weeks after installation of red-foils (red-foil installation date: 25.11.2021); RF0: control; RF1: plants grown under red-foil with transmittance peak of 0.89; B: bufflehead; E: eland; ERF0: Interaction of E and RF0; ERF1: interaction of E and RF1; BRF0: interaction of B and RF0; BRF1: interaction of B and RF1; NS: non-significant; LSD: least significant difference for comparing two treatment means. Values shown are means with standard deviations. Means with different alphabets are significantly different at p<0.05 for each corresponding treatment effect. | | | | | | | | | | | |
